# Global burden of atherosclerotic cardiovascular disease in people with hepatitis C virus infection: a systematic review, meta-analysis, and modelling study

**DOI:** 10.1016/S2468-1253(19)30227-4

**Published:** 2019-07-31

**Authors:** Kuan Ken Lee, Dominik Stelzle, Rong Bing, Mohamed Anwar, Fiona Strachan, Sophia Bashir, David E Newby, Jasmit S Shah, Michael H Chung, Gerald S Bloomfield, Chris T Longenecker, Shashwatee Bagchi, Shyamasundaran Kottilil, Sarah Blach, Homie Razavi, Peter R Mills, Nicholas L Mills, David A McAllister, Anoop S V Shah

**Affiliations:** a British Heart Foundation Centre for Cardiovascular Science, University of Edinburgh, Edinburgh, UK; b Usher Institute of Population Health Sciences and Informatics, University of Edinburgh, Edinburgh, UK; c Department of Neurology, Center for Global Health, Technical University of Munich, Munich, Germany; d Department of Medicine, Aga Khan University, Nairobi, Kenya; e Department of Medicine, Duke Clinical Research Institute and Duke Global Health Institute, Duke University, Durham, NC, USA; f Division of Cardiology, University Hospitals Harrington Heart and Vascular Institute, School of Medicine, Case Western Reserve University, Cleveland, OH, USA; g Division of Infectious Diseases and Institute of Human Virology, University of Maryland School of Medicine, Baltimore, MD, USA; h Center for Disease Analysis Foundation, Lafayette, CO, USA; i Department of Gastroenterology, Gartnavel General Hospital, NHS Greater Glasgow and Clyde, Glasgow, UK; j Institute of Health and Wellbeing, University of Glasgow, Glasgow, UK

## Abstract

**Background:**

More than 70 million people worldwide are estimated to have hepatitis C virus (HCV) infection. Emerging evidence indicates an association between HCV and atherosclerotic cardiovascular disease. We aimed to determine the association between HCV and cardiovascular disease, and estimate the national, regional, and global burden of cardiovascular disease attributable to HCV.

**Methods:**

For this systematic review and meta-analysis, we searched MEDLINE, Embase, Ovid Global Health, and Web of Science databases from inception to May 9, 2018, without language restrictions, for longitudinal studies that evaluated the risk ratio (RR) of cardiovascular disease in people with HCV compared with those without HCV. Two investigators independently reviewed and extracted data from published reports. The main outcome was cardiovascular disease, defined as hospital admission with, or mortality from, acute myocardial infarction or stroke. We calculated the pooled RR of cardiovascular disease associated with HCV using a random-effects model. Additionally, we calculated the population attributable fraction and disability-adjusted life-years (DALYs) from HCV-associated cardiovascular disease at the national, regional, and global level. We also used age-stratified and sex-stratified HCV prevalence estimates and cardiovascular DALYs for 100 countries to estimate country-level burden associated with HCV. This study is registered with PROSPERO, number CRD42018091857.

**Findings:**

Our search identified 16 639 records, of which 36 studies were included for analysis, including 341 739 people with HCV. The pooled RR for cardiovascular disease was 1·28 (95% CI 1·18–1·39). Globally, 1·5 million (95% CI 0·9–2·1) DALYs per year were lost due to HCV-associated cardiovascular disease. Low-income and middle-income countries had the highest disease burden with south Asian, eastern European, north African, and Middle Eastern regions accounting for two-thirds of all HCV-associated cardiovascular DALYs.

**Interpretation:**

HCV infection is associated with an increased risk of cardiovascular disease. The global burden of cardiovascular disease associated with HCV infection was responsible for 1·5 million DALYs, with the highest burden in low-income and middle-income countries.

**Funding:**

British Heart Foundation and Wellcome Trust.

## Introduction

Globally, more than 70 million people are estimated to have hepatitis C virus (HCV) infection.^1^ Prevalence of HCV is particularly high in the eastern Mediterranean region and Europe, where approximately 2·3% and 1·5% of the general population have HCV infection, respectively.^1^ In the USA, the estimated prevalence of past or current HCV infection is 1·4%, affecting 4·6 million people, of whom at least 3·5 million have active HCV infection (1% of the general population).^2^ The number of new incident cases of HCV infections in the USA has been increasing since 2010.^3^

After acute HCV infection, most patients develop chronic infection.^4^ Usually, these patients remain asymptomatic, with less than a third progressing to liver cirrhosis in the subsequent 20–30 years.^4^ Although mortality due to cirrhosis and hepatocellular carcinoma are well recognised long-term complications of chronic HCV infection,^5^, ^6^ patients with chronic infection are also at increased risk of non-liver-related mortality, including cancer and circulatory death.^7^

Atherosclerotic cardiovascular disease is the most common cause of death worldwide and the burden of disease is projected to rise substantially over the next few decades, particularly in low-income and middle-income countries (LMICs).^8^ HCV transmission is also projected to rise considerably in LMICs due to unsafe health-care practices and injection drug use.^9^, ^10^, ^11^ Published data^12^, ^13^, ^14^ suggest that the long period of chronic HCV infection might lead to the development of atherosclerotic cardiovascular disease because of derangements in metabolic pathways and chronic inflammation. However, the direction and strength of the association between HCV infection and cardiovascular disease remains uncertain.^15^, ^16^, ^17^, ^18^, ^19^

**Research in context**

**Evidence before this study**

We searched PubMed from database inception to Jan 1, 2018, for systematic reviews and meta-analyses evaluating the association between hepatitis C virus (HCV) infection and atherosclerotic cardiovascular disease using the search terms “myocardial infarction”, “stroke”, “cerebrovascular disease”, “cardiovascular disease”, and “hepatitis C”. We found no studies that assessed the risk of cardiovascular disease or calculated the burden from all major atherosclerotic cardiovascular events associated with hepatitis C. Previous meta-analyses have evaluated the association between HCV infection and stroke and surrogate markers of subclinical atherosclerotic disease.

**Added value of this study**

To our knowledge, our study is the first meta-analysis to investigate the risk of major atherosclerotic cardiovascular disease in people with HCV infection and to estimate the burden of atherosclerotic cardiovascular disease attributed to HCV infection at the global, regional, and national level.

**Implication of all the available evidence**

Our findings show that people with HCV infection have a higher risk of cardiovascular disease than those without. The global burden of cardiovascular disease attributable to HCV accounted for a substantial number of disability-adjusted life-years in 2015, and the majority of the burden was borne by low-income and middle-income countries. These finding highlights the importance of public health strategies to eradicate HCV infection to reduce the burden of not only hepatic, but extrahepatic complications (such as cardiovascular disease), especially in regions with high HCV prevalence.

Our study aimed to determine the association between HCV infection and the risk of cardiovascular disease to establish the global burden of cardiovascular disease attributable to HCV.

## Methods

### Search strategy and selection criteria

We searched MEDLINE, EMBASE, Ovid Global Health, and Web of Science from database inception to May 9, 2018, for original peer-reviewed articles using the search terms “myocardial infarction”, “stroke”, “cerebrovascular disease”, “cardiovascular disease”, and “hepatitis C” with no language restrictions. Full search terms are in the appendix (pp 2, 3). Additionally, we manually searched relevant review articles and bibliographic reference lists of studies selected for inclusion in our meta-analysis.

We included all longitudinal studies (case-control studies, cohort studies, and randomised controlled trials) that reported risk ratios (RRs) for hospital admission due to atherosclerotic cardiovascular disease or cardiovascular mortality in people with HCV compared with people without HCV. When there were multiple publications using data from the same cohort, we selected the article that reported the longest follow-up period. Detailed full-text review and data extraction was done independently by at least two investigators (KKL, DS, RB, or MA) and any disagreements were resolved by a third investigator (ASVS). We contacted authors for additional data or clarification if required. This study was done in accordance with the Preferred Reporting Items for Systematic Reviews and Meta-Analyses (PRISMA) guidelines (appendix pp 4, 5).^20^ The study protocol is available online.

For studies that stratified the study population according to the presence of HCV RNA viraemia, we used the RR estimates pertaining to individuals who were HCV RNA positive. We defined a cardiovascular event as hospital admission with, or mortality from, acute myocardial infarction or stroke. Studies that evaluated a composite of acute cardiovascular events that included myocardial infarction or stroke but were not exclusive to these conditions were also included. For studies that stratified stroke events into haemorrhagic and ischaemic strokes, we included only ischaemic strokes in the analysis because haemorrhagic strokes have distinct pathophysiological mechanisms that are unrelated to atherosclerosis.^21^

### Data analysis

We extracted RR estimates comparing cardiovascular events in people with HCV versus those without HCV from published reports using a standardised data extraction sheet. We estimated pooled RRs with 95% CIs. Since this outcome was relatively uncommon, we pooled studies that reported odds ratio and RR. We also assumed independence between risk estimates for different endpoints reported within studies, consistent with our previous analysis.^22^ We did a subgroup analysis stratified by outcome, HIV co-infection, publication year, risk of bias, definition of outcome event, and geographical location.

Two independent investigators (KKL and DS) assessed individual studies for risk of bias, using the degree of adjustment for confounders as the primary domain, and any disagreements were adjudicated by a third investigator. Studies that had adjusted for age, sex, and at least one other confounder were classified as being at low risk of bias. Studies that adjusted for fewer confounders than this were classified as moderate or high risk: studies that adjusted for either age or sex without any other confounders were classified as moderate risk of bias and those that did not adjust for both age or sex were classified as high risk of bias. We did the subgroup analysis stratified by risk of bias.

We estimated the burden of cardiovascular disease attributable to HCV at the national, regional, and global level. We obtained 2015 global prevalence estimates of viraemic HCV (HCV RNA positive) for 100 countries from the Polaris Observatory, with estimates stratified by Global Burden of Disease region.^23^ These 100 countries represent more than 85% of the global population and where more than 89% of all HCV viral infections (HCV RNA positive) are estimated to occur worldwide.^23^ The national prevalence estimates obtained were age-specific and sex-specific. We obtained age-specific and sex-specific disability-adjusted life-year (DALY) estimates for cardiovascular disease (DALYs due to ischaemic heart disease and stroke) for all adults aged older than 20 years in 2015 from the Institute of Health Metrics and Evaluation.^24^ The extraction databases from the systematic review and the data from the Polaris Observatory and Institute of Health Metrics and Evaluation used to derive the pooled estimates and the burden estimates alongside the R code script are available online.

We estimated the population attributable risk fraction at the national, regional, and global level using the pooled RR for cardiovascular disease in patients with hepatitis C and the prevalence estimates of HCV. The population attributable fraction (PAF) for cardiovascular disease attributable to HCV was calculated as described previously:^25^, ^26^

$$Populatio\mathrm{n a}ttributabl\mathrm{e f}raction=\frac{Prevalence\times (RR-1)}{1+(Prevalence\times RR)}$$

We then used national, regional, and global level attributable fractions to calculate the burden as previously described (appendix pp 6–10):

DALYs attributable to HCV=Cardiovascular DALYs×PAF

We provided estimates of PAF and burden in 5-year age groups and presented data graphically using a linear model to interpolate the intervening years. We further provided burden estimates by income of nation stratified by high-income versus LMICs. National income status was defined according to the 2018 World Bank classification.^27^

We anticipated heterogeneity in the RRs across studies because of differences in study design, patient population, geographical location, statistical methods, and adjustment for confounders. We pooled RRs using a random effects model to account for within and between study heterogeneity. We assessed heterogeneity in the pooled meta-estimate of the RR using the *I*^2^ statistic. We assessed publication bias using visual inspection of funnel plots of the RR estimates and using Egger's regression test for asymmetry.^28^ We corrected for asymmetry using Duval and Tweedie's trim and fill method.^29^ Full statistical methods are in the appendix (pp 6–10). All analyses were done using R (version 3.4.1). A two-sided p value of less than 0·05 was considered to indicate statistical significance.

### Role of the funding source

The funders of the study had no role in study design, data collection, data analysis, data interpretation, or writing of the report. The corresponding author had full access to all the data in the study and had final responsibility for the decision to submit for publication.

## Results

**Figure 1:**
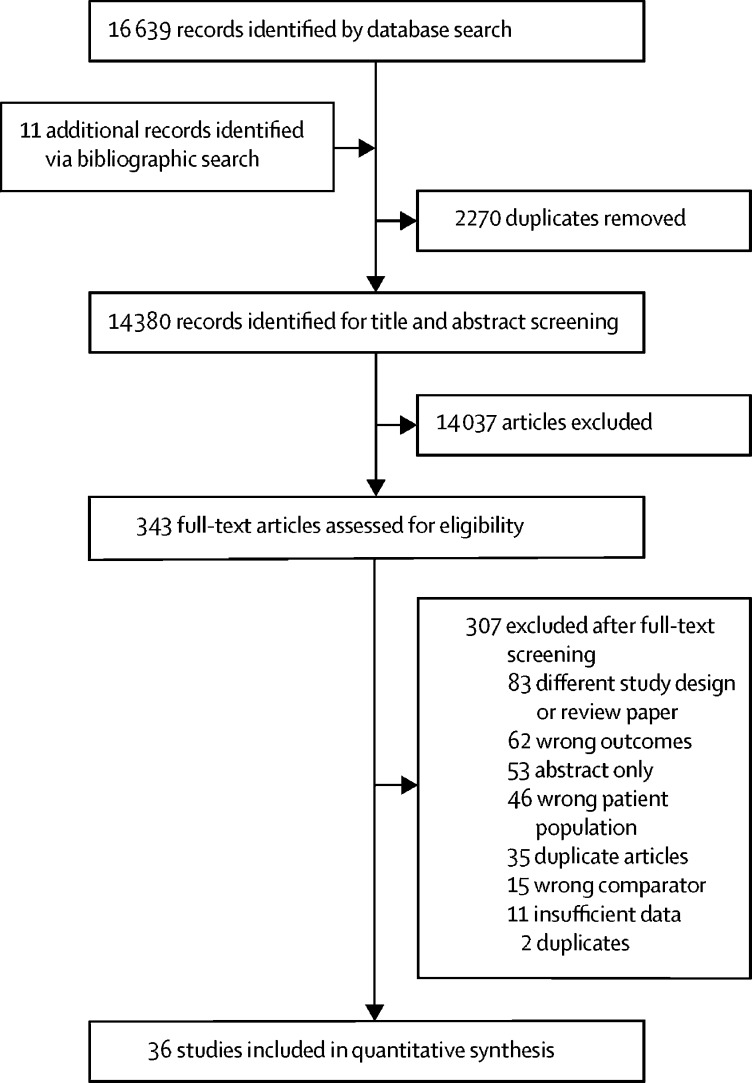
Study selection

We identified 16 650 articles, of which 2270 were duplicates (figure 1). 343 full-text articles were assessed for eligibility. After full-text review, 36 studies,^7^, ^30^, ^31^, ^32^, ^33^, ^34^, ^35^, ^36^, ^37^, ^38^, ^39^, ^40^, ^41^, ^42^, ^43^, ^44^, ^45^, ^46^, ^47^, ^48^, ^49^, ^50^, ^51^, ^52^, ^53^, ^54^, ^55^, ^56^, ^57^, ^58^, ^59^, ^60^, ^61^, ^62^, ^63^, ^64^ which provided 47 estimates, were included in our analyses (table). These studies included 341 739 people with HCV. 31 (86%) of 36 studies were done in North America, Europe, and east Asia. Only two studies^30^, ^31^ originated from LMICs. Most studies used the International Classification of Diseases coding or physician diagnosis to define the outcome events.

**Table:** Baseline characteristics of studies included in the meta-analysis

	**Cohort name**	**Country or region**	**Study type**	**Data source**	**Participants, n**	**Events, n**	**Men, n (%)**	**Mean age at baseline, years**	**Study period**	**Hepatitis C virus status**	**Outcome**	**Outcome definition**
Heo et al, 2018^33^	..	USA	Cohort study	Organ Procurement and Transplant Network	2728	117	1996 (73%)	50·9	2004–14	Seropositive	Cardiovascular disease	Undefined
Alvaro-Meca et al, 2017^34^	..	Spain	Case-control study	Spanish Minimum Basic Data Set	4091	369	3248 (79%)	45	1997–2013	Seropositive	Stroke*	ICD-9
Butt et al, 2017^35^	ERCHIVES	USA	Cohort study	Veterans Health Administration	171 726	5949	171 726 (100%)	54	2001–15	Viraemic	Myocardial infarction	ICD-9
Chew et al, 2017^36^	ERCHIVES	USA	Cohort study	Veterans Health Administration	168 256	11 753	168 256 (100%)	55	2001–14	Seropositive	Cardiovascular disease	ICD-9
Goodkin et al, 2017^30^	DOPPS	Multiple†	Cohort study	Hospital records	76 689	6790	45 016 (59%)	62·5	1996–2015	Seropositive	Cardiovascular disease, myocardial infarction, stroke	Undefined
Kovari et al, 2017^37^	..	Switzerland	Cohort study	Swiss HIV Cohort Study	5006	143	3624 (72%)	50	1994–2014	Seropositive	Cardiovascular disease*	Physician diagnosis
Piazza et al, 2016^38^	..	USA	Cohort study	Hospital records	143	19	101 (71%)	55	2005–10	Undefined	Cardiovascular disease	Undefined
Fernandez-Montero et al, 2015^39^	..	Spain	Cohort study	Hospital register	1066	29	842 (79%)	42·7	2004–15	Viraemic	Cardiovascular disease†	Physician diagnosis
Tsai et al, 2015^40^	NHIRD–HCV	Taiwan	Cohort study	National Health Insurance Research Database	69 915	848	35 936 (51%)	54·7	1998–2008	Undefined	Myocardial infarction	Undefined
Vajdic et al, 2015^41^	..	Australia	Cohort study	Pharmaceutical Drugs of Addiction System	29 571	122	20 403 (69%)	26	1993–2007	Seropositive	Cardiovascular disease	ICD-9, ICD-10
Enger et al, 2014^42^	ORD	USA	Cohort study	Optum Research Database (insurance plans)	90 931	534	56 740 (62%)	49	2000–06	Seropositive	Myocardial infarction, stroke	ICD-9
Gillis et al, 2014^43^	OCS	Canada	Cohort study	Clinic register	4152	167	3483 (84%)	36	1995–2011	Seropositive	Cardiovascular disease†	Physician diagnosis
Hsu et al, 2014^44^	..	Taiwan	Cohort study	National Health Insurance Research Database	7055	429	4599 (65%)	54·9	2003–11	Seropositive	Myocardial infarction, stroke	ICD-9
Pothineni et al, 2014^45^	UAMS	USA	Cohort study	Enterprise Data Warehouse at University of Arkansas for Medical Sciences	23 050	951	12 631 (55%)	50·9	2001–13	Viraemic	Cardiovascular disease	ICD-9
Tripathi et al, 2014^46^	..	USA	Cohort study	Medicaid	13 632	1284	7661 (56%)	38	1994–2011	Seropositive	Cardiovascular disease	ICD-9
Womack et al, 2014^47^	Veterans Aging Cohort Study–virtual cohort	USA	Cohort study	Veterans Health Administration, Medicare, Medicaid, and Quality Enhancement Research Initiative in ischaemic heart disease	2187	86	0	43·6	2003–09	Seropositive	Cardiovascular disease*	ICD-9
Adinolfi et al, 2013^48^	..	Italy	Case-control study	Hospital records	820	123	524 (64%)	76	2010–12	Seropositive	Stroke	Physician diagnosis
Hsu et al, 2013^49^	LHID2000	Taiwan	Cohort study	Longitudinal Health Insurance Database 2000	15 565	NR	8078 (52%)	Not reported	2004–07	Viraemic	Stroke	ICD-9
Younossi et al, 2013^50^	NHANES III	USA	Cohort study	National Health and Nutrition Examination Survey	8985	NR	4178 (46%)	Not reported	1988–2006	Viraemic	Cardiovascular disease	ICD-10
Campbell et al, 2012^51^	..	UK	Cohort study	Hospital records	4068	32	4068 (100%)	36·5	2004–09	Seropositive	Cardiovascular disease*	Physician diagnosis
Carrieri et al, 2012^52^	APROCO-COPILOTE	France	Cohort study	Medical questionnaires	1154	49	900 (78%)	37·7	1997–2010	Seropositive	Cardiovascular disease*	ICD-10
Forde et al, 2012^53^	THIN	UK	Cohort study	General practice medical records	76 477	264	46 727 (61%)	38·6	1996–2008	Undefined	Myocardial infarction	Read diagnostic code
Lee et al, 2012^54^	REVEAL–HCV	Taiwan	Cohort study	Questionnaires and interviews	19 636	477	9523 (48%)	47·6	1991–2008	Viraemic	Cardiovascular disease	ICD-9
Liao et al, 2012^55^	NHIRD	Taiwan	Cohort study	National Health Insurance Research Database	20470	1981	10235 (50%)	52	2002–08	Viraemic	Stroke	ICD-9
Freiberg et al, 2011^56^	Veterans Aging Cohort Study–virtual cohort	USA	Cohort study	Veterans Aging Cohort Study and Large Health Study of Veteran Enrollees	8579	194	8579 (100%)	48·1	2000–07	Seropositive	Cardiovascular disease*	ICD-9
Kristiansen et al, 2011^57^	..	Norway	Cohort study	Department of Microbiology, University Hospital of North Norway	1010	5	686 (68%)	40	1990–2000	Seropositive	Cardiovascular disease	ICD-10
Ohsawa et al, 2011^58^	KAREN	Japan	Cohort study	KAREN cohort	1077	194	682 (63%)	60·4	2003–08	Seropositive	Cardiovascular disease	ICD-10
Bedimo et al, 2010^59^	HIV Clinical Care Registry	USA	Cohort study	Veterans Registry	19 424	1146	18 938 (97%)	46·2	1984–2004	Viraemic	Myocardial infarction, stroke*	ICD-9
Belloso et al, 2010^31^	LATINA	Brazil, Mexico	Cohort study	LATINA cohort	160	40	Not reported	Not reported	1997–2007	Seropositive	Cardiovascular disease*	Physician diagnosis
DAD Study Group, 2010^60^	DAD study	Europe, USA, Australia	Cohort study	DAD cohort	21 815	517	16 143 (74%)	38	1999–2007	Seropositive	Myocardial infarction*	WHO MONICA Project
Lee et al, 2010^61^	..	Taiwan	Cohort study	National Death Certification Registry	23 665	22	11 879 (50%)	47·1	1991–92	Viraemic	Stroke	ICD-9
Tsui et al, 2009^32^	The Heart and Soul study	USA	Cohort study	Veterans Administration electronic records	981	151	803 (82%)	66·3	2000–06	Seropositive	Cardiovascular disease	Physician diagnosis
Guiltinan et al, 2008^62^	..	USA	Cohort study	Blood Systems	20 518	88	13 254 (65%)	Not reported	1991–2002	Seropositive	Cardiovascular disease	ICD-9 CM, ICD-10 CM
Kalantar-Zadeh et al, 2007^63^	..	USA	Cohort study	DaVita outpatient dialysis database	13 664	NR	7433 (54%)	60·1	2001–04	Seropositive	Cardiovascular disease	Undefined
Arcari et al, 2006^64^	..	USA	Case-control study	Clinical registry	75 834	292	47 775 (63%)	40·2	1991–2000	Seropositive	Myocardial infarction	ICD-9
Amin et al, 2006^7^	..	Australia	Cohort study	New South Wales Health Department Notifiable Diseases Database	582	450	582 (100%)	34	1990–2002	Seropositive	Cardiovascular disease	ICD-9 and ICD-10

ICD=International Classification of Diseases. ERCHIVES=Electronically Retrieved Cohort of Hepatitis C Virus Infected Veterans. DOPPS=Dialysis Outcomes and Practice Patterns Study. NHIRD–HCV=National Health Insurance Research Database–Hepatitis C Virus. ORD=Optum Research Database. OCS=Ontario HIV Treatment Network Cohort Study. UAMS=University of Arkansas for Medical Sciences. LHID2000=Longitudinal Health Insurance Database 2000. NR=not reported. NHANES III=National Health and Nutrition Examination Survey III. APROCO-COPILOTE=Antiprotéases Cohorte. THIN=The Health Improvement Network. REVEAL–HCV=Risk Evaluation of Viral Load Elevation and Associated Liver Disease/Cancer–Hepatitis C Virus. DAD=Data Collection on Adverse Events of Anti-HIV Drugs. MONICA=Multinational Monitoring of Trends and Determinants in Cardiovascular Disease. CM=Clinical Modification.

* Risk ratio reported for hepatitis C virus and HIV co-infection versus HIV infection only.

† Australia, Belgium, Canada, mainland China, France, Germany, Bahrain, Kuwait, Oman, Qatar, Saudi Arabia, United Arab Emirates, Italy, Japan, New Zealand, Spain, Russia, Sweden, Turkey, the UK, and the USA.

**Figure 2:**
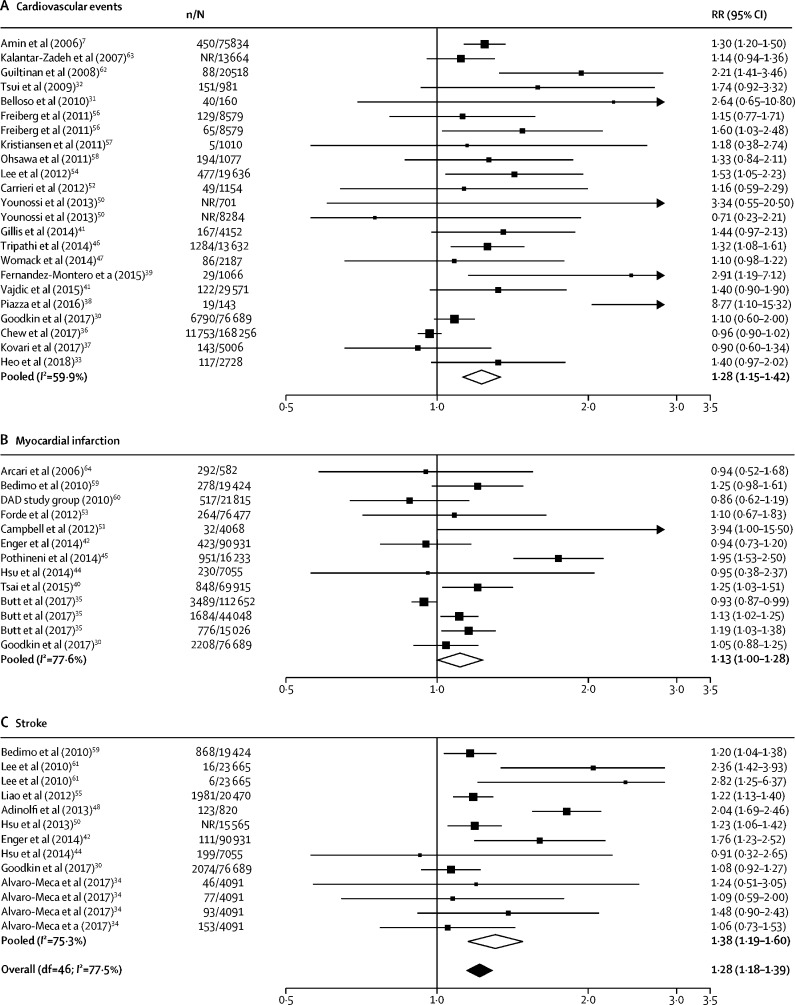
Forest plots of pooled RRs for cardiovascular disease in people with hepatitis C virus versus people without Pooled RRs for composite cardiovascular events (A), myocardial infarction (B), and stroke (C). n=number of events. N=number of participants. RR=risk ratio. NR=not reported. df=degrees of freedom. DAD=Data Collection on Adverse Events of Anti-HIV Drugs.

24 studies defined HCV infection as anti-HCV antibody seropositivity, nine studies used detectable HCV RNA levels, and three studies did not explicitly define their approach (table). Overall, the meta-analysis showed that individuals with HCV had a higher risk of cardiovascular disease than individuals without HCV (pooled RR 1·28, 95% CI 1·18–1·39; figure 2). When stratified by outcome the risk ratio was 1·13 (95% CI 1·00–1·28) for myocardial infarction, 1·38 (1·19–1·60) for stroke, and 1·39 (1·24–1·55) for cardiovascular mortality (appendix p 11). Individuals with HCV and HIV co-infection had a higher risk of cardiovascular disease than those with HIV mono-infection (RR 1·20, 1·09–1·32). Post-hoc analyses showed that the RR estimates from studies published before 2014, which was the median publication year, were marginally higher than those from studies published after 2014 (1·39 [1·25–1·54] *vs* 1·22 [1·11–1·34]). Studies that ascertained outcome with physician diagnosis had higher RRs than did those that used International Classification of Diseases codes (1·68 [1·24–2·29] *vs* 1·31 [1·20–1·42]). Nine studies in patients with HCV viraemia had marginally higher RRs than the overall pooled RR (1·32, 1·15–1·51). Nearly two-thirds of studies originated from the USA or Taiwan (21 [58%] of 36 studies). In the subgroup analysis, studies from these two countries had a similar pooled RR as those from the rest of the world (1·28 [1·16–1·40] *vs* 1·29 [1·12–1·48] respectively).

There was significant heterogeneity (*I*^2^=77·5%) and publication bias in the overall estimate (Egger's test p=0·003). Using the trim and fill method to correct for funnel plot asymmetry did not change the direction of effect but did attenuate the effect size (appendix p 20). 11 studies were at moderate or high risk of bias (appendix pp 12, 13). Compared with studies with a low risk of bias, those with moderate or high risk of bias had a similar pooled RR (1·30 [95% CI 1·10–1·55] for studies with moderate or high risk of bias *vs* 1·29 [1·19–1·40] for studies with low risk of bias).

We estimated that in 2015, 1·5 million (95% CI 0·9–2·1) DALYs from cardiovascular disease were attributable to HCV, with marked geographical variation in the estimated burden. LMICs had the highest disease burden, with South Asia, eastern Europe, north Africa, and the Middle East accounting for nearly two-thirds of the global burden of cardiovascular disease attributable to HCV in 2015 (920·7 thousand DALYs; appendix p 14).

**Figure 3:**
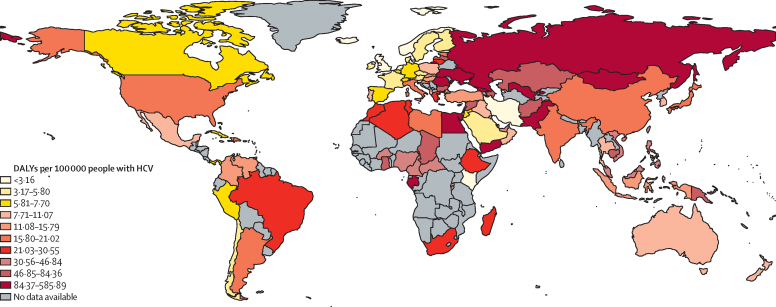
DALYs per 100 000 people for cardiovascular disease attributable to HCV Grey colour denotes regions for which no HCV prevalence data were available to estimate burden. HCV=hepatitis C virus. DALYs=disability-adjusted life-years.

**Figure 4:**
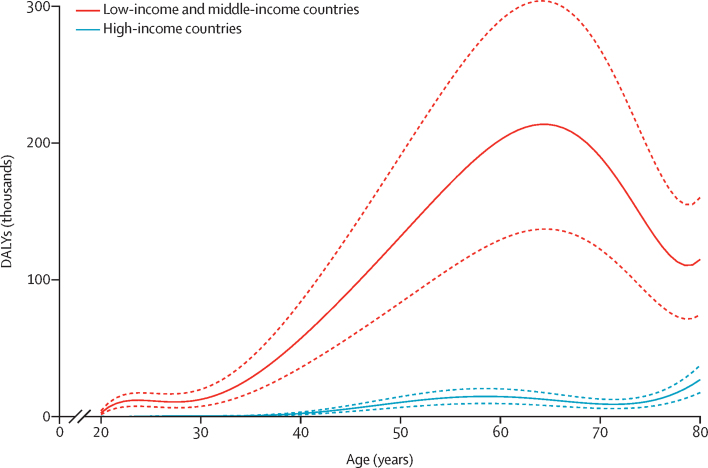
DALYs for cardiovascular disease attributable to hepatitis C virus Solid lines show the central estimate and dashed lines show the 95% CI. DALYs=disability-adjusted life-years.

Of the 100 countries with available age-specific and sex-specific viraemic HCV prevalence estimates for 2015, the highest burden (ie, cardiovascular DALYs attributable to HCV) was in Ukraine, Mongolia, Gabon, and Egypt (figure 3; appendix pp 15–17). Worldwide, the PAF of cardiovascular disease attributable to HCV was highest in people aged 55–59 years (appendix p 18). DALYs from cardiovascular disease attributable to HCV was highest in people aged 70–74 years (appendix p 18). The burden of cardiovascular disease attributable to HCV was higher in LMICs than in high-income countries (1·4 million [95% CI 0·88–1·95] DALYs *vs* 0·1 million [95% CI 0·07–0·12] DALYs; figure 4; appendix p 19).

## Discussion

In this systematic review and meta-analysis, we assessed the association between HCV and cardiovascular disease and estimated the global, regional, and national burden of cardiovascular disease attributable to HCV. We made several key observations. First, people with HCV have an increased risk of cardiovascular disease compared with those without HCV (RR 1·28). When stratified by type of cardiovascular event, the overall pooled estimate was higher for stroke than for myocardial infarction. Second, our pooled risk estimate was derived from 341 739 people with HCV infection included in 36 studies from 51 countries. Only two studies^30^, ^31^ reported findings from populations of LMICs, highlighting the paucity of data from these regions. Third, the most up-to-date annual global burden of cardiovascular disease attributable to HCV was 1·5 million DALYs. Most of this burden was concentrated in the 55–75 year age group, reflecting more premature development of cardiovascular disease in people with HCV. Fourth, considerable geographical variation was identified in the burden of cardiovascular disease attributable to HCV, with the highest burden observed in south Asia, eastern Europe, north Africa, and the Middle East. The majority of the burden was borne by LMICs rather than high-income countries. This observation is likely to reflect both a high prevalence of chronic hepatitis C in these regions and an increasing burden of cardiovascular disease.

Our analysis has several strengths. We included longitudinal studies that evaluated the association between HCV and hospital admissions with, or mortality from, cardiovascular disease. Furthermore, the endpoint of our analysis was major adverse cardiovascular events, which enabled accurate risk estimation and assessment of cardiovascular burden. Previous systematic reviews and meta-analyses,^65^, ^66^, ^67^ which included cross-sectional studies and studies that used surrogate endpoints that might not be fully reflective of a causal relationship, have reported divergent findings. We also analysed burden using age-specific, sex-specific, and country-specific cardiovascular burden and HCV prevalence estimates, allowing us to provide HCV attributable burden estimates for specific age groups, which could be useful for policy makers. Moreover, our estimates for HCV prevalence obtained from the Polaris Observatory^23^ reflect viraemia rather than just seropositivity alone, and the countries for which we had prevalence estimates accounted for over 89% of all chronic HCV infections globally. Our estimates for the PAF and subsequent HCV associated cardiovascular burden are therefore based on a high-risk population with active HCV infection, in whom both long-term hepatic and extrahepatic complications remain common.

This study has a number of limitations. Most studies included in this meta-analysis originated from high-income countries in North America and western Europe, but estimates were applied to all regions. This approach is commonly used in this type of analysis because of paucity of data from LMICs.^68^, ^69^ This highlights an ongoing need for research in these low-resource settings, in which the disease prevalence of HCV and cardiovascular disease is high, to improve the accuracy of the burden estimates in these regions. We also observed significant heterogeneity in our RR estimates. However, the direction of effect was consistent and robust across all subgroup analyses. The observed heterogeneity is likely to reflect the diverse patient population, viraemic status of the study population, differences in health-care systems, access to treatment, and geographical location of the studies pooled in this analysis. Many studies did not fully account for the competing risk of non-cardiovascular mortality, thus some methodological heterogeneity exists. Most people with HCV infection die from non-cardiovascular causes,^7^, ^70^ therefore this is an important competing risk that might distort the exposure–outcome association with cardiovascular disease. People with HIV and HCV co-infection have a higher risk for cardiovascular disease than those with HIV mono-infection. This increased risk highlights the importance of risk stratification in this patient population considering that people with HIV are twice as likely to have cardiovascular events than those without HIV.^22^ Although most studies evaluating the RR of cardiovascular disease adjusted for risk factors for cardiovascular disease, a substantial possibility of residual confounding remains. Furthermore, there was substantial publication bias in the literature, which might have influenced the risk estimates. However, there was little attenuation of the RRs when analysis was restricted to studies without moderate to high risk of bias or after accounting for publication bias using the trim and fill method. Additionally, we pooled RR estimates of myocardial infarction or stroke to estimate the PAF and combined this with the DALYs for ischaemic heart disease and cerebrovascular disease to estimate the burden of cardiovascular disease attributable to HCV. We were unable to estimate the burden of angina or peripheral artery disease attributable to HCV since these conditions are often diagnosed in the outpatient setting and are less likely to be captured by electronic health record systems. Therefore, it is possible that we have underestimated the cardiovascular burden associated with HCV. However, the 2010 Global Burden of Disease study^24^, ^71^ showed that angina and peripheral artery disease contributed a relatively small proportion of the overall cardiovascular disease burden. The studies included in this meta-analysis are likely to be exposed to a degree of outcome misclassification bias because most studies used routine diagnostic coding to define cardiovascular events rather than clinical adjudication. All of the included studies were observational studies, and thus we are unable to establish causality.

The underlying pathophysiological mechanism for the association between HCV and cardiovascular disease remains unclear.^15^ HCV infection has been associated with conditions such as type 2 diabetes, a well known cardiovascular risk factor.^72^ Evidence has emerged showing direct effects of HCV on the development of atherosclerosis,^73^ beyond that attributable to metabolic derangements alone. Chronic HCV infection results in a chronic state of immune stimulation and inflammation evidenced by increased circulating levels of proinflammatory cytokines, such as interleukin 6, tumour necrosis factor-α, C-reactive protein, and fibrinogen, all of which are associated with the development of atherosclerotic cardiovascular disease.^32^, ^74^, ^75^ Interferon-based antiviral treatments for HCV reduce markers of inflammation, endothelial dysfunction, and diabetes mellitus.^76^, ^77^, ^78^ Sustained viral response with direct-acting antivirals have also been associated with a lower risk of cardiovascular events.^79^ Whether eradication of HCV infection reduces future risk of adverse cardiovascular events should be further explored in randomised controlled trials of direct-acting antivirals to investigate this finding.

The link between HCV and cardiovascular disease has important implications for the formulation of health policies and resource allocation, particularly in regions with limited health-care resources, where chronic HCV infection remains prevalent and cardiovascular disease burden is increasing. Globally, prevalence of HCV is projected to increase substantially, particularly in LMICs, as a result of transmission via unsafe health-care related injections and injection drug use.^9^, ^10^, ^11^ Consequently, mortality due to hepatic and extrahepatic complications of HCV is likely to increase considerably if efforts to improve early testing and treatment are not implemented. A disproportionate burden of cardiovascular disease is borne by LMICs, where currently more than 80% of the global burden is concentrated.^80^ Our estimates suggest that more than 90% of the global cardiovascular burden attributable to HCV occurs in LMICs.

The introduction of direct-acting antiviral therapies with the ability to achieve sustained virological response in more than 90% of treated individuals should be a cause for optimism. These new therapeutic options enable the prevention of both hepatic and extrahepatic complications of HCV infection with a shorter duration of treatment and fewer adverse events than previous generations of antiviral therapies. However, at present, public health programmes and access to health-care services for people with HCV lags behind other comparable infectious diseases, such as HIV or malaria.^1^ The provision of direct-acting antiviral therapies in patients with HCV infection remains low on the global scale, with only one in 15 patients^1^ currently being treated, the majority of whom reside in high-income countries.^81^, ^82^ To have the greatest impact on HCV morbidity and mortality, the delivery of curative HCV treatment needs to be coupled with efficient health systems to provide chronic care services for patients with both hepatic and extrahepatic complications of HCV. Considering our study findings, investment in greater strategic integration and linkage of viral hepatitis services with other relevant services, including cardiovascular disease prevention, might be a cost-effective method of facilitating the prevention and management of concurrent major health conditions. These innovative approaches to health-care delivery might require further research to evaluate feasibility and efficacy in a real-world clinical setting.

People with HCV have a higher risk of developing cardiovascular disease than those without HCV. HCV accounted for 1·5 million DALYs due to cardiovascular disease worldwide in 2015, with the highest burden in South Asia, eastern Europe, north Africa, and the Middle East. Most of the disease burden is borne by LMICs, where HCV prevalence is projected to rise substantially. Our findings are of public health importance and could inform future research and health-care policies to improve risk stratification and treatment strategies aimed at reducing the combined global burden of HCV and extrahepatic sequelae such as cardiovascular disease.

For the **extraction databases, data used to derive pooled estimates, burden estimates, and R code script** see https://github.com/kk-lee/hcv
