# Supplementary material for: Global burden of atherosclerotic cardiovascular disease in people with hepatitis C virus infection: a systematic review, meta-analysis, and modelling study
Source: Lancet Gastroenterol Hepatol. 2019 Jul 31;4(10):794–804. doi: 10.1016/S2468-1253(19)30227-4 (PMC6734111; doi:10.1016/S2468-1253(19)30227-4)

THE LANCET
Gastroenterology & Hepatology

Supplementary appendix

This appendix formed part of the original submission and has been peer reviewed. We post it as supplied by the authors.

Supplement to: Lee KK, Stelzle D, Bing R, et al. Global burden of atherosclerotic cardiovascular disease in people with hepatitis C virus infection: a systematic review, meta-analysis, and modelling study. *Lancet Gastroenterol Hepatol* 2019; published online July 31. http://dx.doi.org/10.1016/S2468-1253(19)30227-4.

SUPPLEMENTARY MATERIAL

Global burden of atherosclerotic cardiovascular disease in people living with the hepatitis C virus: *A systematic review, meta-analysis and modelling study.*

Kuan Ken Lee MD*[1], Dominik Stelzle MD*[2], Rong Bing MD[1], Mohamed Anwar MD[1], Fiona Strachan PhD[1], Sophia Bashir BSc[1], Prof David E Newby MD[1], Jasmit S Shah PhD[3], Prof Michael H Chung MD[3], Gerald S Bloomfield MD[4], Chris T Longenecker MD[5], Shashwatee Bagchi MD[6], Prof Shyamasundaran Kottilil MD[6], Sarah Blach MHS[7], Homie Razavi PhD[7], Prof Peter R Mills MD[8], Prof Nicholas L Mills MD[1], David A McAllister MD[9] and Anoop SV Shah MD[1,10]

[1]*BHF Centre for Cardiovascular Science, University of Edinburgh*
[2] *Center for Global Health, Department of Neurology, Technical University, Munich*
[3]*Department of Medicine, Aga Khan University*
[4]*Department of Medicine, Duke Clinical Research Institute and Duke Global Health Institute, Duke University*
[5]*Division of Cardiology, University Hospitals Harrington Heart and Vascular Institute, Case Western Reserve University School of Medicine*
[6]*Division of Infectious Diseases and Institute of Human Virology, University of Maryland School of Medicine*
[7]*Center for Disease Analysis Foundation, Lafayette, Colorado, USA*
[8]*Department of Gastroenterology, Gartnavel General Hospital, NHS Greater Glasgow and Clyde, Glasgow*
[9]*Institute of Health and Wellbeing, University of Glasgow*
[10]*Usher Institute of Population Health Sciences and Informatics, University of Edinburgh*
**Contributed equally*

Correspondence and requests for reprints:
Dr Anoop SV Shah
BHF/University Centre for Cardiovascular Science,
SU.305 Chancellor's Building,
University of Edinburgh,
Edinburgh EH16 4SB
United Kingdom
Tel: +44 131 242 6432
Fax: +44 131 242 6379
Mobile: +44 7766 544156
E-mail: Anoop.Shah@ed.ac.uk

Prospero registration: CRD42018091857
Table and Figures: 7
Support: British Heart Foundation Clinical Research Training Fellowship (FS/18/25/33454), Senior Clinical Research Fellowship (FS/16/04/32023), Chair Award (CH/09/002) and Centre of Excellence Award (RE/13/3/30183), Wellcome Trust Intermediate Clinical Fellowship (201492/Z/16/Z) and Senior Investigator Award (WT103782AIA).

Supplementary Text 1: Search criteria

Medline- Ovid MEDLINE 1946 to 9th May 2018
Embase- Ovid EMBASE 1974 to 9th May 2018
Global Health- 1910 to 9th May 2018
Web of Science- 1946 to 9th May 2018

Medline:

1. exp hepatitis, chronic/ or exp hepatitis, viral, human/
2. exp Hepatitis C/ or HCV.mp. or exp Hepatitis C, Chronic/
3. exp Flavivirus Infections/ or exp Flaviviridae Infections/ or exp Flaviviridae/
4. antiviral.mp. or exp Antiviral Agents/
5. Ribavirin.mp. or exp Ribavirin/
6. Interferon.mp. or exp Interferons/
7. Sofosbuvir.mp. or exp Sofosbuvir/
8. 1 or 2 or 3 or 4 or 5 or 6 or 7
9. exp Myocardial Infarction/
10. exp Coronary Thrombosis/
11. Cardiovascular Disease.mp.
12. acute coronary.mp.
13. exp Angina, Unstable/
14. Myocardial infarct$.mp.
15. heart infarct.mp.
16. acs.mp.
17. ami.mp.
18. (coronary adj3 syndrome$).mp.
19. acute angina.mp.
20. (unstable adj3 angina).mp.
21. unstable coronary.mp.
22. cerebrovascular disorders/ or basal ganglia cerebrovascular disease/ or brain ischemia/ or exp brain infarction/ or hypoxia-ischemia, brain/ or carotid artery diseases/ or carotid artery thrombosis/ or carotid artery, internal, dissection/ or intracranial arterial diseases/ or cerebral arterial diseases/ or infarction, anterior cerebral artery/ or infarction, middle cerebral artery/ or infarction, posterior cerebral artery/ or exp "intracranial embolism and thrombosis"/ or exp stroke/ or vertebral artery dissection/
23. (isch?emi$ adj5 (stroke$ or apoplex$ or cerebral vasc$ or cerebrovasc$ or cva)).tw.
24. ((brain or cerebr$ or cerebell$ or vertebrobasil$ or hemispher$ or intracran$ or intracerebral or infratentorial or supratentorial or middle cerebr$ or mca$ or anterior circulation or basilar artery or vertebral artery) adj5 (isch?emi$ or infarct$ or thrombo$ or emboli$ or occlus$ or hypoxi$)).tw.
25. 10 or 11 or 12 or 13 or 14 or 15 or 16 or 17 or 18 or 19 or 20 or 21 or 22 or 23 or 24 or 25
26. 8 and 25
27. limit 26 to humans

Embase

1. hepatitis.mp. or exp hepatitis/
2. hcv.ti. or hcv.ab.
3. exp flaviviridae/
4. exp pegylated interferon/ or exp peginterferon/ or exp lamivudine/ or exp adefovir/ or exp telbivudine/ or exp entecavir/
5. exp sofosbuvir/ or exp dasabuvir/ or exp simeprevir/ or exp grazoprevir/ or exp daclatasvir/ or exp ledipasvir/ or exp ombitasvir/ or exp velpatasvir/ or exp elbasvir/ or antiviral therapy.mp. or antiviral agent.mp.
6. 1 or 2 or 3 or 4 or 5
7. exp Heart Infarction/
8. coronary thrombosis.mp. or exp Coronary Artery Thrombosis/
9. cardiovascular disease.mp.
10. acute coronary.mp.
11. exp Unstable Angina Pectoris/

12. Myocardial infarct$.mp.
13. heart infarct:.mp.
14. acs.mp.
15. ami.mp.
16. (coronary adj3 syndrome$).mp.
17. acute angina.mp.
18. (unstable adj3 angina).mp.
19. brain infarction/ or brain stem infarction/ or cerebellum infarction/ or exp brain ischemia/ or carotid artery disease/ or exp carotid artery obstruction/ or cerebral artery disease/ or exp cerebrovascular accident/ or exp occlusive cerebrovascular disease/ or stroke patient/
20. (isch?emi$ adj5 (stroke$ or apoplex$ or cerebral vasc$ or cerebrovasc$ or cva)).tw.
21. ((brain or cerebr$ or cerebell$ or vertebrobasil$ or hemispher$ or intracran$ or intracerebral or infratentorial or supratentorial or middle cerebr$ or mca$ or anterior circulation or basilar artery or vertebral artery) adj5 (isch?emi$ or infarct$ or thrombo$ or emboli$ or occlus$ or hypoxi$)).tw.
22. 7 or 8 or 9 or 10 or 11 or 12 or 13 or 14 or 15 or 16 or 17 or 18 or 19 or 20 or 21
23. 6 and 22
24. limit 23 to humans

Global health

1. brain infarction.mp. [mp=abstract, title, original title, broad terms, heading words, identifiers, cabicodes]
2. brain stem infarction.mp. [mp=abstract, title, original title, broad terms, heading words, identifiers, cabicodes]
3. stroke.mp. or exp stroke/
4. exp infarction/
5. carotid artery disease.mp.
6. carotid artery obstruction.mp.
7. cerebrovascular accident.mp. or cerebrovascular disorders.sh.
8. exp Myocardial Infarction/
9. acute coronary syndrome.mp. or cardiovascular diseases.sh. or myocardial ischaemia.sh.
10. acs.mp.
11. ami.mp.
12. (unstable adj3 angina).mp.
13. acute angina.mp.
14. unstable angina.mp.
15. 1 or 2 or 3 or 4 or 5 or 6 or 7 or 8 or 9 or 10 or 11 or 12 or 13 or 14
16. hepatitis.ti. or hepatitis.ab. or hcv.ab. or hcv.ti. or hepatitis C virus.ti. or hepatitis C virus.ab. or flaviviridae.ti. or flaviviridae.ab.
17. antiviral.mp. or exp Antiviral Agents/
18. Ribavirin.mp. or exp Ribavirin/
19. Interferon.mp. or exp Interferons/
20. Sofosbuvir.mp. or exp Sofosbuvir/
21. 16 or 17 or 18 or 19 or 20
22. 15 and 21

Web of science

1. TS=(Myocardial Infarction OR Coronary Thrombosis OR acute coronary syndrome OR ACS OR unstable angina OR cerebrovascular disease OR stroke OR brain infarction OR brain ischaemia OR brain ischemia OR cardiovascular disease)

2. TS = (hepatitis OR hepatitis C virus OR flaviviridae OR ritonavir OR interferon OR sofosbuvir OR lamivudine OR perginterferon OR ribavirin OR dasabuvir OR simeprevir OR grazoprevir OR daclatasvir OR ledipasvir OR ombitasvir OR velpatasvir OR elbasvir OR anti-hepatitis agents OR antiviral agents OR anti-viral agents)

3. 1 and 2

Supplementary Text 2: PRISMA checklist

Section/topic	#	Checklist item	Reported on page #
**TITLE**			
Title	1	Identify the report as a systematic review, meta-analysis, or both.	1
**ABSTRACT**			
Structured summary	2	Provide a structured summary including, as applicable: background; objectives; data sources; study eligibility criteria, participants, and interventions; study appraisal and synthesis methods; results; limitations; conclusions and implications of key findings; systematic review registration number.	1, 2, 3
**INTRODUCTION**			
Rationale	3	Describe the rationale for the review in the context of what is already known.	4
Objectives	4	Provide an explicit statement of questions being addressed with reference to participants, interventions, comparisons, outcomes, and study design (PICOS).	3, 5
**METHODS**			
Protocol and registration	5	Indicate if a review protocol exists, if and where it can be accessed (e.g., Web address), and, if available, provide registration information including registration number.	1
Eligibility criteria	6	Specify study characteristics (e.g., PICOS, length of follow-up) and report characteristics (e.g., years considered, language, publication status) used as criteria for eligibility, giving rationale.	6, 7
Information sources	7	Describe all information sources (e.g., databases with dates of coverage, contact with study authors to identify additional studies) in the search and date last searched.	6
Search	8	Present full electronic search strategy for at least one database, including any limits used, such that it could be repeated.	supplement
Study selection	9	State the process for selecting studies (i.e., screening, eligibility, included in systematic review, and, if applicable, included in the meta-analysis).	6, 7
Data collection process	10	Describe method of data extraction from reports (e.g., piloted forms, independently, in duplicate) and any processes for obtaining and confirming data from investigators.	6, 7
Data items	11	List and define all variables for which data were sought (e.g., PICOS, funding sources) and any assumptions and simplifications made.	6, 7 table 1
Risk of bias in individual studies	12	Describe methods used for assessing risk of bias of individual studies (including specification of whether this was done at the study or outcome level), and how this information is to be used in any data synthesis.	7, supplement

Summary measures	13	State the principal summary measures (e.g., risk ratio, difference in means).	6, 7
Synthesis of results	14	Describe the methods of handling data and combining results of studies, if done, including measures of consistency (e.g., I^2) for each meta-analysis.	7
Risk of bias across studies	15	Specify any assessment of risk of bias that may affect the cumulative evidence (e.g., publication bias, selective reporting within studies).	7, supplement
Additional analyses	16	Describe methods of additional analyses (e.g., sensitivity or subgroup analyses, meta-regression), if done, indicating which were pre-specified.	7, 8, 9
**RESULTS**			
Study selection	17	Give numbers of studies screened, assessed for eligibility, and included in the review, with reasons for exclusions at each stage, ideally with a flow diagram.	10, supplement
Study characteristics	18	For each study, present characteristics for which data were extracted (e.g., study size, PICOS, follow-up period) and provide the citations.	table 1
Risk of bias within studies	19	Present data on risk of bias of each study and, if available, any outcome level assessment (see item 12).	supplement
Results of individual studies	20	For all outcomes considered (benefits or harms), present, for each study: (a) simple summary data for each intervention group (b) effect estimates and confidence intervals, ideally with a forest plot.	figure 1
Synthesis of results	21	Present results of each meta-analysis done, including confidence intervals and measures of consistency.	figure 1
Risk of bias across studies	22	Present results of any assessment of risk of bias across studies (see Item 15).	10, supplement
Additional analysis	23	Give results of additional analyses, if done (e.g., sensitivity or subgroup analyses, meta-regression [see Item 16]).	10, supplement
**DISCUSSION**			
Summary of evidence	24	Summarize the main findings including the strength of evidence for each main outcome; consider their relevance to key groups (e.g., healthcare providers, users, and policy makers).	12
Limitations	25	Discuss limitations at study and outcome level (e.g., risk of bias), and at review-level (e.g., incomplete retrieval of identified research, reporting bias).	12, 13
Conclusions	26	Provide a general interpretation of the results in the context of other evidence, and implications for future research.	14, 15, 16
**FUNDING**			
Funding	27	Describe sources of funding for the systematic review and other support (e.g., supply of data); role of funders for the systematic review.	1

Supplementary Text 3: Method for calculating country specific estimates for cardiovascular disease attributable to HCV

Step 1
We used a random-effects model (DerSimonian and Laird method) to account for significant within and between study heterogeneity. We extracted risk ratios for cardiovascular disease in people living with HCV versus those without from the relevant studies. The risk ratios were then pooled using a random effects generalised linear mixed model with the rma.glmm function in the metaphor package in R.

Step 2
We obtained age- and sex-specific estimates of HCV prevalence (adults >20 years) in 2015 for 100 countries from the Polaris Observatory. Similarly, we obtained age- and sex-specific estimates of cardiovascular disability-adjusted life years in 2015 from the global burden of disease estimates for these countries.

Step 3
Assumptions:

- We assumed that the pooled risk ratios obtained from a range of case-control and cohort studies were applicable across both developed and developing countries.
- The degree of adjustment for confounders varied between the primary studies and therefore estimates may be limited by residual confounding.

Step 4
We estimated the population attributable fraction and DALYs as follows:

$$Population\ Attributable\ Fraction\ (PAF) = \frac{Prevalence * \ (RR - 1)}{1 + Prevalence * (RR - 1)}$$

$$DALYs\ attributable\ to\ HCV = Cardiovascular\ DALYs \times PAF$$

We estimated the uncertainty intervals for the DALYs attributable to HCV and PAF using simulation. We sampled from the probability distributions which captured the uncertainty around the estimate for each component (specifically, log normal for rate ratios, normal for the cardiovascular disability-adjusted life years and beta for the prevalence) calculating the age- and sex-specific PAF and DALYs for each of 10,000 samples. Total DALYs for each country were reported with uncertainty estimates as 95% confidence interval. The uncertainty ranges can be viewed as approximate Bayesian posterior probability limits with very diffuse priors for the estimates and risk factor rate ratios.

The schematic below illustrates the steps involved to derive the cardiovascular DALYs due to HCV for Ukraine.

Deriving the age- and sex-specific population attributable fraction of Cardiovascular disease attributable to HCV

Ukraine (Men, age 70 – 74 years)

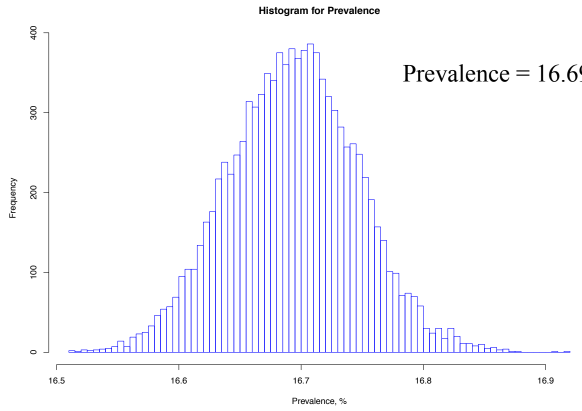

Prevalence = 16.69% (16.59% – 16.80%)

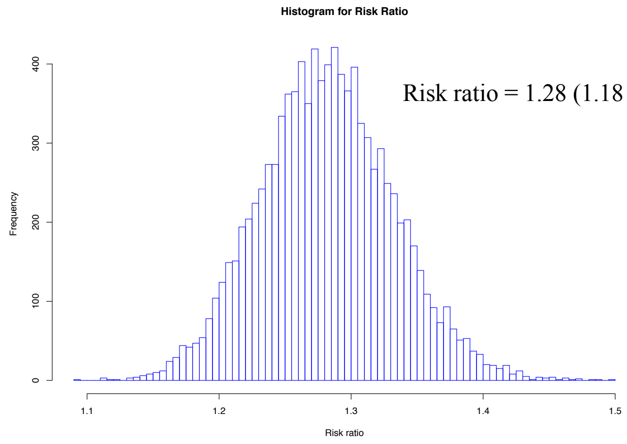

Risk ratio = 1.28 (1.18 – 1.39)

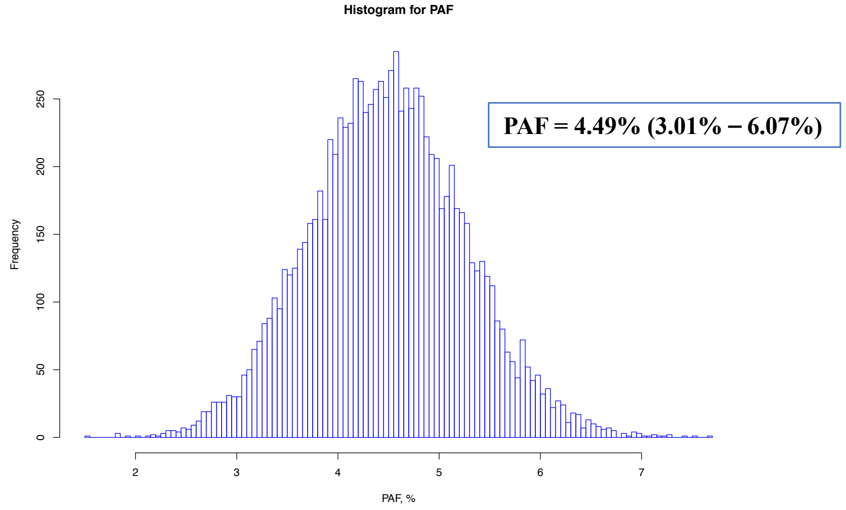

PAF = 4.49% (3.01% – 6.07%)

$$Population\ Attributable\ Fraction\ (PAF) = \frac{Prevalence * (RR - 1)}{1 + Prevalence * (RR - 1)}$$

Deriving the age- and sex-specific DALYs of Cardiovascular disease attributable to HCV

Ukraine (Men, age 70 – 74 years)

PAF = 4.49% (3.01% – 6.07%)

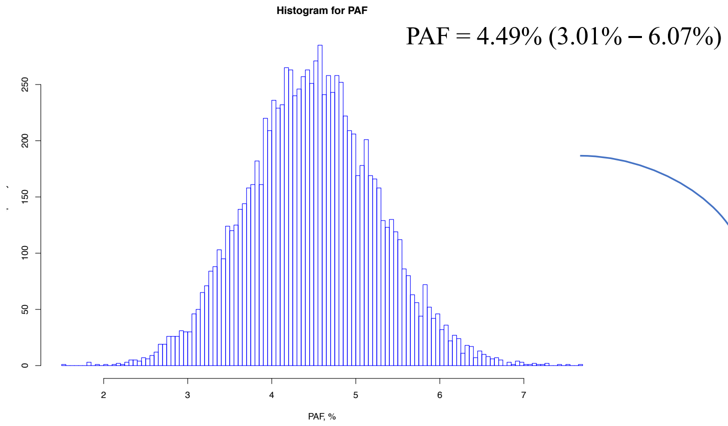

CV DALYs = 810 (757 – 867)

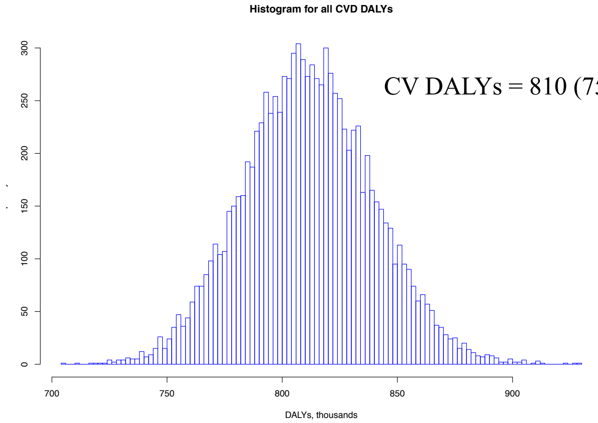

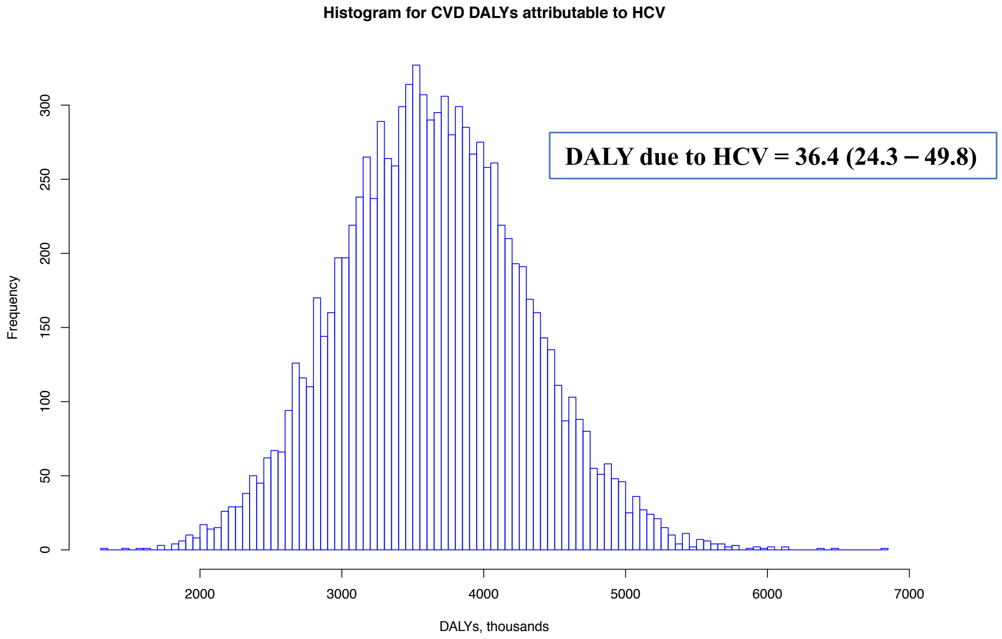

$$DALYs\ attributable\ to\ HCV = Cardiovascular\ DALYs \times PAF$$

Calculating total CVD DALYs attributable to HCV in Ukraine

Age group, years	Gender	Total Population	HCV positive	Prevalence of HCV, %	Total CV DALYs, thousands	PAF, %	CVD DALYs attributable to HCV, thousands
**20-24**	female	1271000	50200	3·95	3·08 (2·32-4·06)	1·1 (0·73-1·5)	0·03 (0·02-0·05)
	male	1329000	51300	3·86	6·93 (5·05-9·05)	1·07 (0·71-1·47)	0·07 (0·05-0·11)
**25-29**	female	1775000	72400	4·08	5·87 (4·4-7·53)	1·13 (0·75-1·55)	0·07 (0·04-0·1)
	male	1842000	74900	4·07	18·86 (14·15-24·16)	1·13 (0·75-1·55)	0·21 (0·13-0·32)
**30-34**	female	1831000	91200	4·98	9·62 (7·19-12·39)	1·38 (0·92-1·89)	0·13 (0·08-0·2)
	male	1877000	99500	5·3	34·59 (26·48-44·48)	1·47 (0·98-2·01)	0·51 (0·31-0·77)
**35-39**	female	1743000	118000	6·77	17·67 (13·72-22·75)	1·87 (1·25-2·55)	0·33 (0·2-0·49)
	male	1686000	124000	7·35	59·04 (46·23-73·49)	2·03 (1·35-2·76)	1·19 (0·76-1·77)
**40-44**	female	1643000	132000	8·03	22·59 (17·72-28·2)	2·21 (1·47-3·01)	0·5 (0·31-0·74)
	male	1531000	138000	9·01	76·25 (61·72-91·83)	2·47 (1·65-3·37)	1·88 (1·2-2·75)
**45-49**	female	1479000	146000	9·87	49·05 (39·7-58·98)	2·7 (1·8-3·68)	1·32 (0·85-1·93)
	male	1330000	145000	10·9	159·1 (135·42-185·82)	2·98 (1·99-4·04)	4·73 (3·06-6·72)
**50-54**	female	1769000	176000	9·95	94·41 (79·48-111)	2·72 (1·82-3·7)	2·56 (1·66-3·65)
	male	1486000	179000	12·05	271·92 (236·84-310·16)	3·28 (2·19-4·45)	8·89 (5·88-12·46)
**55-59**	female	1747000	199000	11·39	179·42 (157·59-202·45)	3·11 (2·07-4·22)	5·57 (3·66-7·7)
	male	1332000	190000	14·26	389·53 (347·79-434·2)	3·86 (2·58-5·23)	15·04 (9·9-20·81)
**60-64**	female	1915000	189000	9·87	304·53 (273·42-337·06)	2·7 (1·8-3·67)	8·23 (5·41-11·38)
	male	1312000	160000	12·2	573·17 (523·48-625·16)	3·32 (2·22-4·5)	19·02 (12·58-26·16)
**65-69**	female	1178000	153000	12·99	205·73 (187·68-224·1)	3·53 (2·36-4·78)	7·24 (4·81-10)
	male	733000	113000	15·42	251·59 (231·59-272·18)	4·16 (2·79-5·62)	10·44 (6·97-14·3)
**70-74**	female	993000	125000	12·59	850·09 (783·45-917·12)	3·42 (2·29-4·64)	29·07 (19·29-39·74)
	male	502000	83800	16·69	810·45 (756·55-867·44)	4·49 (3·01-6·07)	36·41 (24·31-49·78)

**75-79**	female	1201000	120000	9·99	344·99 (319·84-370·21)	2·73 (1·82-3·72)	9·44 (6·23-12·9)
	male	548000	74900	13·67	268·09 (250·87-286·89)	3·7 (2·48-5·02)	9·94 (6·64-13·56)
**80+**	female	1142000	90800	7·95	1070·87 (1010·52-1131·56)	2·19 (1·46-2·98)	23·39 (15·54-32·07)
	male	418000	41300	9·88	449·84 (425·74-476·11)	2·71 (1·8-3·68)	12·16 (8·09-16·64)
**Total**		35613000	3137300	8·81	6527·6 (5957·9-7128·8)		208·38 (137·98-287·11)

Supplementary Table 1: Sensitivity and subgroup analysis for the risk ratio

	Number of estimates	Risk ratio	Lower limit	Upper limit	Heterogeneity, I^2 (%)
**Overall**	47	1·28	1·18	1·39	77·5
**Outcome**					
Incidence	33	1·25	1·14	1·37	83·3
Mortality	21	1·39	1·24	1·55	49·4
**HIV co-infection**					
HCV/HIV co-infected[a]	15	1·20	1·09	1·32	18·1
**Publication year**[b]					
Pre 2014	28	1·39	1·25	1·54	64·8
Post 2014	26	1·22	1·11	1·34	81·4
**Risk of bias**[c]					
Low	41	1·29	1·19	1·40	74·1
Moderate / High	13	1·30	1·10	1·55	78·6
**Definition of outcome event**					
ICD diagnostic code	36	1.31	1.20	1.42	79.2
Physician diagnosis	7	1.68	1.24	2.29	56.4
**Countries/territories**					
Studies from USA or Taiwan	29	1.28	1.16	1.40	80.3
Studies not from USA or Taiwan	18	1.29	1.12	1.48	63.2

[a] HCV/HIV co-infected patients versus HIV mono-infected patients
[b] Median publication year was 2014
[c] Risk of bias assessed by level of adjustment. Low risk – adjustment of age, sex and at least one other covariate
Abbreviations: HCV- hepatitis C virus, HIV- human immunodeficiency virus, USA- United States of America.

Supplementary Table 2: Risk of bias in studies providing risk ratio estimate

Author, publication year	Adjusted Confounders			Risk of Bias
	Age	Sex	Other Confounders	
**Heo et al, 2018**	Yes	Yes	BMI, African-American, diabetes, hypertension, time on haemodialysis, primary kidney disease, donor factors	Low
**Alvaro-Meca et al, 2017**	Yes	Yes	Tobacco use and Charlson comorbidity index	Low
**Butt et al, 2017**	Yes	Yes	Ethnicity, BMI, lipid lowering therapy, hypertension, diabetes, smoking, COPD, CKD	Low
**Chew et al, 2017**	No	No		High
**Goodkin et al, 2017**	Yes	Yes	Time on dialysis, Hep B infection, albumin, phosphorus, creatinine, hypertension, diabetes, heart failure, cerebrovascular disease, cancer, neurologic disorder, peripheral vascular disease, cirrhosis	Low
**Kovari et al, 2017**	Yes	No	HIV acquisition category, smoking, alcohol use, active intravenous drug use, and duration of HIV and HCV infection	Moderate
**Piazza et al, 2016**	Yes	Yes	Smoking, NASH vs alcoholic cirrhosis, MELD score, FHx heart disease, BMI, history of heart disease	Low
**Fernandez-Montero et al, 2015**	Yes	Yes	Virological parameters, smoking, hypertension, diabetes, LDL	Low
**Tsai et al, 2015**	Yes	Yes	Hypertension, diabetes, hyperlipidaemia, stroke, COPD, heart failure, Hepatitis B, cirrhosis.	Low
**Vajdic et al, 2015**	No	No		High
**Enger et al, 2014**	Yes	Yes	Hypertension, past and recent steroid use	Low
**Gillis et al, 2014**	Yes	Yes	Ethnicity, year of ART initiation, weight and baseline smoking status	Low
**Hsu et al, 2014**	Yes	Yes	Demographic factors, comorbidity, diabetes medication, hypertension, dyslipidaemia, chronic obstructive lung disease and peripheral arterial occlusive disease	Low
**Pothineni et al, 2014**	Yes	Yes	Hypertension, diabetes, COPD	Low
**Tripathi et al, 2014**	Yes	Yes	Diabetes, smoking, hypertension, dyslipidaemia, obesity, hepatitis B, race	Low
**Womack et al, 2014**	Yes	Yes	Diabetes, smoking, hypertension, dyslipidaemia, race, renal disease, anemia, BMI, cocaine, alcohol, statins	Low
**Adinolfi et al, 2013**	No	Yes	Smoking, diabetes, hypertension, atrial fibrillation	Moderate
**Hsu et al, 2013**	Yes	Yes	Hypertension, diabetes, hyperlipidaemia, IHD, alcohol related illness, COPD, aspirin use, Clopidogrel use, warfarin use, dipyridamole use, ticlopidine use, statin use, ACEI use and influenza vaccination	Low
**Younossi et al, 2013**	Yes	Yes	Diabetes, smoking, hypertension, race, obesity	Low
**Campbell et al, 2012**	Yes	Yes	Black ethnicity, HIV, renal function, cholesterol, antiretroviral therapy	Low
**Carrieri et al, 2012**	No	No		High

**Forde et al, 2012**	Yes	Yes	Hypertension, diabetes, hyperlipidaemia, family history, smoking, chronic kidney disease, BMI, baseline aspirin use	Low
**Liao et al, 2012**	Yes	Yes	Hyperlipidaemia, diabetes, hypertension, heart disease, lower income, less urbanised area	Low
**Lee et al, 2012**	Yes	Yes	Cigarette smoking, alcohol drinking, betel nuts chewing, central obesity, personal history of diseases and baseline serum levels of cholesterol and triglycerides	Low
**Freiberg et al, 2011**	Yes	No	Race/ethnicity, education, body mass index, hypertension, diabetes, smoking, hypercholesterolemia, HIV viral load, CD4 count, history of alcohol dependence and abuse and cocaine dependence and abuse, and death	Moderate
**Kristiansen et al, 2011**	Yes	Yes		Moderate
**Ohsawa et al, 2011**	Yes	Yes	Diabetes, smoking, hypertension, dyslipidaemia, BMI, history of MI, stroke, malignancy, alcohol, hypotension, elevated CRP, hypoalbuminemia	Low
**Bedimo R. et al, 2010**	Yes	No	Hypertension, type 2 diabetes and tobacco use	Moderate
**Belloso et al, 2010**	No	No		High
**DAD Study Group, 2010**	Yes	Yes	HIV transmission risk, ethnicity, previous AIDS, smoking status, family history of cardiovascular disease (CVD), body mass index, cumulative exposure to non-nucleoside/nucleotide reverse transcriptase inhibitors, protease inhibitors and nucleoside/nucleotide reverse transcriptase inhibitors, previous CVD, cohort, development of diabetes mellitus and calendar year	Low
**Lee et al, 2010**	Yes	Yes	Cigarette smoking status, habitual alcohol consumption, serum triglycerides and cholesterol, BMI, diabetes, hypertension, and heart disease	Low
**Tsui et al, 2009**	Yes	Yes	Race, smoking, diabetes, hypertension, physical activity, statin use, ACE inhibitor/ARB use, total cholesterol, HDL, IL-6, CRP	Low
**Guiltinan et al, 2008**	No	No		High
**Kalantar-Zadeh et al, 2007**	Yes	Yes	Race and ethnicity (African American and other self-categorized black, non-Hispanic white, Asian, Hispanic, and other), diabetes, ischemic heart disease, congestive heart failure, status post cardiac arrest, status post myocardial infarction, pericarditis, cardiac dysrhythmia, cerebrovascular events, peripheral vascular disease, chronic obstructive pulmonary disease, and cancer, categories of dialysis vintage (6 mo, 6 mo to 2 yr, 2 to 5 yr, and 5 yr), primary insurance (Medicare, Medicaid, private, and other), marital status (married, single, divorced, widowed, and other or unknown), the standardized mortality ratio of the dialysis clinic during entry quarter, dialysis dosage as indicated by Kt/V (single pool), presence or absence of a dialysis catheter, and residual renal function during the entry quarter (i.e., urinary urea clearance), 13 surrogates of nutritional status and inflammation, including BMI, the average dosage of rHuEPO, and 11 laboratory variables as surrogates of the nutritional state or inflammation	Low
**Arcari et al, 2006**	Yes	No	Ethnicity, education level, and marital status	Moderate
**Amin et al, 2006**	Yes	Yes	Calendar year	Low

Supplementary Table 3: Burden of HCV associated with cardiovascular disease stratified by Global Burden of Disease regions

Region	CVD DALYs attributable to HCV, thousands
Andean Latin America	1·3 (0·8-1·9)
Australasia	1·7 (1·1-2·4)
Caribbean	2·3 (1·5-3·4)
Central Asia	47·6 (30·3-68·8)
Central Europe	31·8 (20·7-44·5)
Central Latin America	14·2 (9·2-19·7)
Central Sub-Saharan Africa	4·5 (2·1-9·4)
East Asia	191 (123·8-265)
Eastern Europe	304·3 (198·4-420·2)
Eastern Sub-Saharan Africa	16·1 (7·3-34·6)
High-income Asia Pacific	22·9 (14·9-31·6)
High-income North America	42·5 (27·8-58·4)
North Africa and Middle East	243·9 (153·3-359·7)
Oceania	2·4 (1·2-4·5)
South Asia	374·7 (235·1-550·1)
Southeast Asia	91·2 (54·8-142·2)
Southern Latin America	6·4 (4·1-9)
Southern Sub-Saharan Africa	9·6 (6·2-13·7)
Tropical Latin America	39·3 (25·5-54·2)
Western Europe	23·9 (15·5-33·7)
Western Sub-Saharan Africa	47·1 (22·4-97·2)

Supplementary Table 4: National estimates of age and sex stratified disability-adjusted life years for HCV associated with cardiovascular disease (using prevalence of HCV and CVD burden for adults >20 years)

Country	Total Population	HCV positive	HCV prevalence, %	Total CVD DALYs per 100,000 persons (95%CI)	CVD DALYs attributable to HCV per 100,000 persons (95%CI)
**Afghanistan**	14947000	143820	0·96	16056·66 (10073·72-23825·73)	61 (34-105·1)
**Albania**	2122800	8650	0·41	7542·47 (6223·69-8982·67)	10·3 (6·4-15·2)
**Algeria**	25797300	335200	1·3	4211·39 (3244·67-5331·1)	27·9 (17·4-41·6)
**American Samoa**	102070	189	0·19	2042·55 (1477·73-2748·6)	1·2 (0·5-2·6)
**Argentina**	29128000	325090	1·12	4470·11 (3912·81-5072·43)	19·5 (12·6-27·5)
**Australia**	17983000	228710	1·27	2893·32 (2559·28-3252·28)	7·7 (5-10·9)
**Austria**	6905000	21059	0·30	4054·4 (3636·77-4517·03)	3·2 (2·1-4·6)
**Azerbaijan**	6975900	181661	2·60	8547·37 (7280·51-9998·9)	46·8 (29·9-67·8)
**Bahrain**	997640	16535	1·66	1924·87 (1439·88-2528·14)	14·8 (9-23)
**Belgium**	8689000	63920	0·74	3785·29 (3285·36-4316·57)	7 (4·5-10)
**Brazil**	141229000	1793800	1·27	4563·83 (4195·7-4987·38)	27·8 (18·3-38·6)
**Bulgaria**	5746000	83480	1·45	13960·48 (12532·9-15467·96)	65·2 (42·7-91·5)
**Burkina Faso**	8147800	230380	2·83	3368·72 (1597·7-6632·3)	30·5 (13·4-69·1)
**Burundi**	5222100	107940	2·07	3697·41 (1653·12-7155·43)	40·3 (18·2-88)
**Cambodia**	9623400	249800	2·60	5815·79 (4158·31-7822·93)	83·6 (51-129·5)
**Cameroon**	11228600	149160	1·33	4417·12 (1994·77-9063·85)	42·6 (19·1-93·8)
**Canada**	28084000	209500	0·75	3100·32 (2730·72-3496·43)	7·3 (4·7-10·2)
**Central African Republic**	2514200	14630	0·58	8378·66 (2394·78-17558·3)	34·7 (13-94·3)
**Chad**	5987300	141600	2·37	4051·3 (1617·35-9020·41)	57·3 (23·9-138·1)
**Chile**	13217000	55913	0·42	2977·2 (2546·69-3448·36)	5·4 (3·5-7·7)
**China**	1067334000	9594000	0·90	5642·33 (5189·56-6124·26)	17·1 (11·3-23·8)
**Colombia**	32979000	390300	1·18	2743·27 (2377·59-3138·15)	13·5 (8·8-19·1)
**Croatia**	3391000	25750	0·76	8493·19 (7598·44-9437·2)	12·6 (8·1-18)
**Cuba**	8880500	32720	0·37	5715·57 (5029·08-6446·73)	7 (4·5-9·9)
**Czech Republic**	8578800	41220	0·48	7032·44 (6360·72-7740·95)	3·5 (2·3-5·1)
**Denmark**	4392400	19340	0·44	3641·81 (3170·93-4158·55)	3·2 (2-4·6)
**Dominican Republic**	6515300	67130	1·03	5058·04 (4309·32-5898·93)	19·9 (12·7-28·6)
**Egypt**	54600000	5518000	10·11	6981·08 (5804·74-8441·15)	341·1 (221·3-487·2)
**Estonia**	1007800	17960	1·78	8473·17 (7480·6-9570·05)	15·8 (9·9-23·5)
**Ethiopia**	47960500	615300	1·28	3997·14 (2004·4-7711·32)	23 (10·5-49·3)
**Fiji**	564690	529	0·09	9734·02 (7244·91-12802·46)	2·7 (1·4-5·1)
**Finland**	4342700	22520	0·52	4946·38 (4375·86-5579·45)	3·6 (2·3-5·2)
**France**	49069000	188600	0·38	2914·03 (2534·93-3331·74)	3·6 (2·3-5)

**Gabon**	931900	111440	11·96	4599·23 (2393·91-8568·75)	380·7 (189·9-753·9)
**Georgia**	3050300	162820	5·34	13278·48 (11361·09-15481·69)	167·6 (107·4-241)
**Germany**	66349000	204290	0·31	4913·09 (4394·2-5473·66)	5·8 (3·8-8·1)
**Ghana**	14313900	385616	2·69	4903·5 (2241·45-10030·85)	47·4 (19·9-111·7)
**Greece**	9258000	131550	1·42	6168·17 (5512·42-6854·12)	23 (15-32·4)
**Hungary**	7926000	51330	0·65	8467·39 (7538·53-9449·46)	14·5 (9·4-20·6)
**Iceland**	250500	1096	0·44	2727·74 (2391·09-3090·26)	2 (1·1-3·4)
**India**	826348000	6200100	0·75	6524·98 (5967·82-7165·02)	19·6 (12·9-27·4)
**Indonesia**	164471000	1170130	0·71	7260·04 (5126·09-9688·97)	15·7 (9·4-24·9)
**Iran**	55524000	172144	0·31	5193·77 (3776·16-6957·12)	2·8 (1·6-4·6)
**Iraq**	18302900	57480	0·31	7820·52 (5200·73-11123·97)	8·3 (4·7-13·9)
**Ireland**	3454200	30349	0·88	2996·05 (2588·59-3435·87)	4 (2·5-5·8)
**Israel**	5273300	98110	1·86	2328·84 (2027·06-2652·3)	11·2 (7·3-16)
**Italy**	49717000	691405	1·39	4038·21 (3558·39-4544·98)	17·6 (11·5-24·8)
**Japan**	104417000	855730	0·82	3611·64 (3387·43-3840·35)	19 (12·5-26·2)
**Jordan**	4248900	23980	0·56	2748·02 (2094·4-3496·13)	6·1 (3·7-9·2)
**Kazakhstan**	11890600	452870	3·81	10437·65 (8953·99-12053·45)	81·7 (52·4-117·2)
**Kenya**	22673100	109810	0·48	2134·03 (1680·12-2643·39)	2 (1·3-3)
**Latvia**	1584500	42360	2·67	12821·38 (11528·04-14187·44)	56·4 (36·4-80·4)
**Lebanon**	3678700	7460	0·2	4866·58 (3169·35-6846·48)	5·1 (2·9-8·5)
**Libya**	3937300	36230	0·92	4382·81 (3241·81-5794·79)	17·5 (10·7-27)
**Lithuania**	2366100	31830	1·35	11050·71 (10008·39-12178·93)	28·7 (18·5-40·9)
**Luxembourg**	446800	5258	1·18	2556·14 (2226·63-2912·25)	5·9 (3·7-8·9)
**Madagascar**	11823200	55190	0·47	5932·41 (2406·24-11697·27)	21·1 (9·4-47·1)
**Malaysia**	20568200	376720	1·83	4921·23 (3740·36-6298·91)	34·7 (21·4-52·8)
**Malta**	347100	1226	0·35	3897·79 (3452·3-4372·89)	2·4 (1·3-4)
**Mexico**	78271000	526700	0·67	2899·56 (2741·17-3064·55)	8·5 (5·6-11·8)
**Mongolia**	1942900	178090	9·17	9833·04 (7817·56-12145·62)	439·9 (282·7-636·9)
**Morocco**	22436400	249500	1·11	4106·38 (2678·78-6068·25)	29·3 (17-48·1)
**Netherlands**	13039000	34770	0·27	3136·79 (2722·76-3581·89)	1·6 (1-2·3)
**New Zealand**	3409200	47246	1·39	3272·44 (2892·1-3681·86)	8·3 (5·3-11·8)
**Nigeria**	85398100	2286800	2·68	2218·47 (1288·76-4244·06)	34·4 (17·5-66·3)
**Norway**	3899000	20630	0·53	3086·39 (2716·74-3491·27)	2·4 (1·5-3·4)
**Oman**	3089200	14857	0·48	2253·25 (1637·35-2985·61)	7·8 (4·7-12·2)
**Pakistan**	109125000	6291500	5·77	8297·94 (6132·91-10999·88)	194·8 (119·5-298·2)
**Panama**	2570700	12190	0·47	2828·69 (2214·99-3552·32)	6·4 (4-9·7)
**Papua New Guinea**	4087200	79860	1·95	9824·25 (5558·68-16265·12)	59·2 (30·5-111·5)
**Peru**	20217400	164650	0·81	1862·97 (1435·48-2377·71)	6·4 (4-9·6)
**Philippines**	58727800	596800	1·02	6512·2 (5449·14-7709·71)	34·9 (22·4-49·8)
**Poland**	30916000	180360	0·58	6756·95 (6050·81-7517·03)	10·5 (6·8-14·7)
**Portugal**	8640900	87890	1·02	3988·3 (3490·8-4511·2)	8·4 (5·4-12)

**Puerto Rico**	2726600	34780	1·28	3880·27 (3343·9-4477·46)	14·9 (9·5-21·3)
**Qatar**	1812930	36850	2·03	1093·81 (763·11-1523·38)	15·8 (9·4-25·4)
**Romania**	17161000	528100	3·08	10410·99 (9290·73-11600·18)	130·6 (85·6-182·7)
**Russia**	111830000	4576700	4·09	14779·66 (13358·68-16294·27)	84·3 (54·8-119·2)
**Saudi Arabia**	20467000	95530	0·47	2624·6 (2234·54-3094·27)	4·7 (3-6·7)
**Slovakia**	4397900	31990	0·73	6932·69 (6108·89-7822·27)	11·1 (7·1-15·9)
**Slovenia**	1685700	6124	0·36	4152·92 (3598·9-4736·19)	1·9 (1·2-3)
**South Africa**	31891500	354850	1·11	4608·15 (3975·29-5352·36)	30·2 (19·5-43)
**South Korea**	39873000	227500	0·57	2653·46 (2276·08-3065·49)	7·9 (5·1-11·2)
**Spain**	37184000	383956	1·03	2979·66 (2616·89-3371·33)	7·5 (4·8-10·6)
**Sweden**	7519100	36820	0·49	4258·24 (3844·9-4694·73)	3·8 (2·5-5·3)
**Switzerland**	6415600	75670	1·18	2771·21 (2433·29-3138·62)	7·1 (4·6-10·1)
**Syria**	9705000	390330	4·02	6728·41 (5050·29-8564·75)	57·4 (35·3-87·8)
**Taiwan**	18797000	484400	2·58	2919·36 (2204·27-3783·17)	44·3 (27·5-66·9)
**Thailand**	51944000	458130	0·88	4086·02 (2906·55-5539·28)	9·8 (5·8-15·9)
**The Gambia**	887090	16883	1·9	3052·17 (1711·42-5265·76)	22 (10·9-43·4)
**Tunisia**	7892000	93602	1·19	3604·36 (2517·66-4980·98)	21·2 (12·6-33·9)
**Turkey**	52707000	486800	0·92	3277·72 (2690·41-3941·27)	11·5 (7·3-16·7)
**Ukraine**	35613000	3137300	8·81	18329·2 (16729·62-20017·36)	585·1 (387·5-806·2)
**United Arab Emirates**	7781950	126040	1·62	3361·38 (2132·05-5014·55)	45·2 (25·9-76·1)
**United Kingdom**	49276000	162730	0·33	3864·96 (3598·05-4136·13)	2·3 (1·5-3·2)
**United States**	240274000	2927107	1·22	4161·14 (3933·76-4391·23)	16·9 (11·1-23·2)
**Uzbekistan**	19059400	1233110	6·47	7789·36 (6444·7-9205·79)	110 (70·1-161)
**Venezuela**	19966500	115290	0·58	4138·08 (3366·83-5015·79)	14·2 (9·1-20·6)
**Vietnam**	66598000	1065200	1·6	4546·32 (2944·87-6421·98)	37 (21·2-61·8)
**Yemen**	13473200	205240	1·52	8123·36 (4499·82-13878·5)	88·3 (45·9-165·3)

[a] Age- and sex-stratified prevalence of HCV in all 100 countries are available on our online repository

Supplementary Table 5: Burden of HCV associated with cardiovascular disease stratified by age groups

Age groups, years	Total Population	HCV positive	HCV prevalence, %	Population attributable fraction, %	DALYs, thousands
**20-24**	507846300	2591751	0·51	0·14 (0·09-0·2)	3·54 (2·15-5·5)
**25-29**	532239200	4024487	0·76	0·21 (0·14-0·29)	8·07 (4·93-12·47)
**30-34**	492962000	5108935	1·04	0·29 (0·19-0·4)	16·96 (10·46-25·87)
**35-39**	443058900	5929922	1·34	0·37 (0·25-0·51)	31·1 (19·44-46·56)
**40-44**	433873600	6580239	1·52	0·42 (0·28-0·58)	54·1 (33·95-80·42)
**45-49**	415267000	6994876	1·68	0·47 (0·31-0·65)	95·85 (60·9-140·43)
**50-54**	371960600	7110809	1·91	0·53 (0·35-0·73)	145·49 (93·19-210·39)
**55-59**	316021900	6718961	2·13	0·59 (0·39-0·82)	188·6 (121·21-271·46)
**60-64**	275369300	5501557	2·00	0·56 (0·37-0·77)	224·33 (145·19-320·02)
**65-69**	206980000	4020861	1·94	0·54 (0·36-0·75)	196·93 (127·59-280·81)
**70-74**	144432400	2632881	1·82	0·51 (0·34-0·7)	228·41 (149·11-321·64)
**75-79**	106871740	1681154	1·57	0·44 (0·29-0·6)	135·06 (88·01-190·97)
**80+**	123239630	1527367	1·24	0·35 (0·23-0·48)	144·08 (94·51-200·7)

Supplementary Table 6: Disability-adjusted life years for HCV associated cardiovascular disease stratified by high-income versus low- and middle-income countries

Age groups, years	DALYs, thousands	
	High-income countries	Low- and Middle-income countries
20-24	0·05 (0·03-0·08)	3·49 (2·12-5·42)
25-29	0·13 (0·08-0·2)	7·94 (4·85-12·27)
30-34	0·34 (0·21-0·5)	16·62 (10·25-25·37)
35-39	0·78 (0·49-1·15)	30·32 (18·95-45·41)
40-44	1·99 (1·27-2·89)	52·11 (32·68-77·53)
45-49	5·64 (3·63-8·04)	90·21 (57·27-132·39)
50-54	10·65 (6·92-15·01)	134·84 (86·27-195·38)
55-59	13·59 (8·9-19·01)	175 (112·31-252·45)
60-64	15·2 (9·99-21·16)	209·13 (135·2-298·86)
65-69	11·02 (7·24-15·34)	185·91 (120·35-265·47)
70-74	10·09 (6·61-14·06)	218·32 (142·5-307·58)
75-79	10·96 (7·19-15·26)	124·1 (80·81-175·72)
80+	27·02 (17·78-37·44)	117·06 (76·73-163·27)

Supplementary Figure 1: Funnel plot

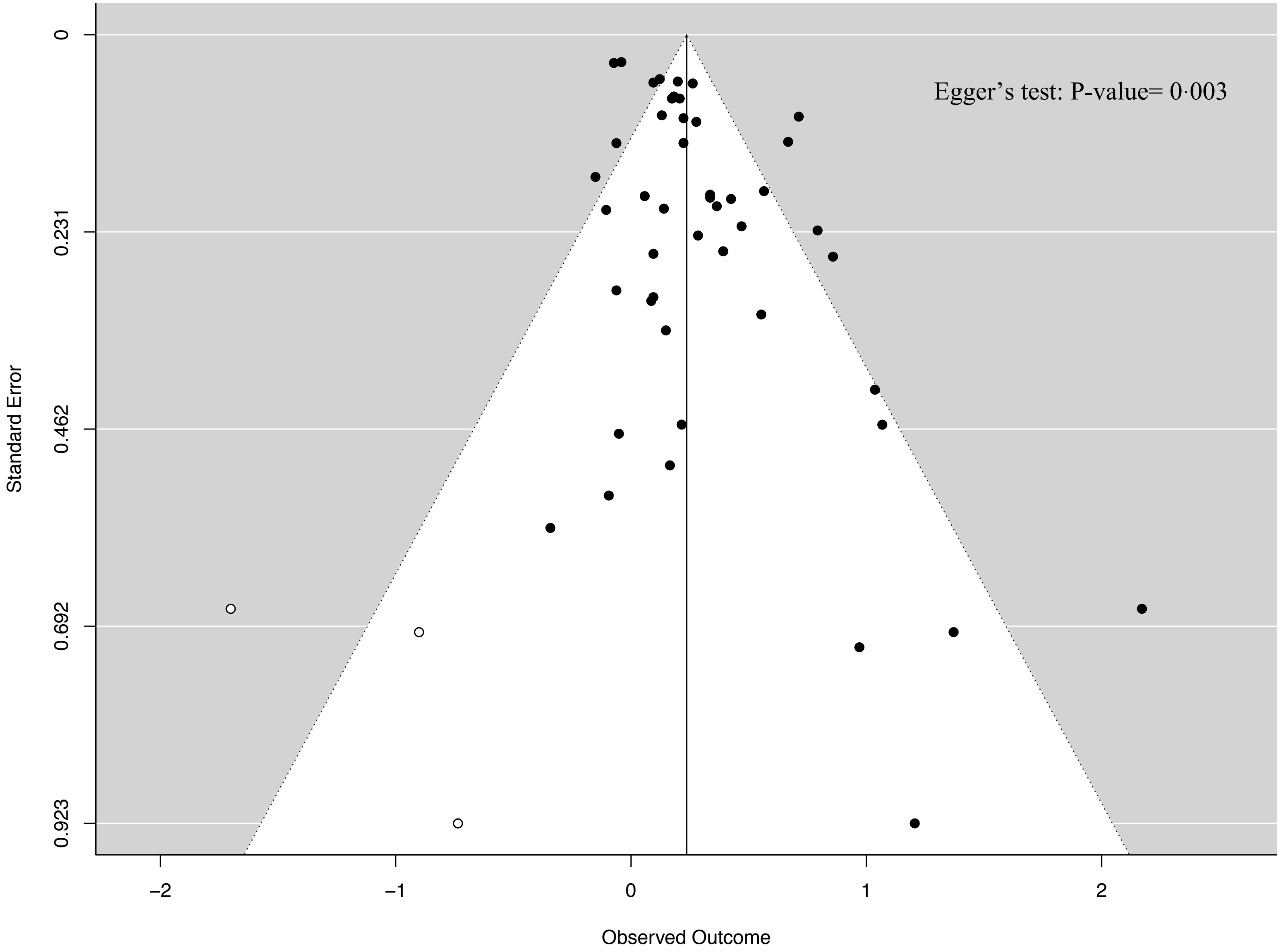

Supplement: Supplementary appendix [file mmc1.pdf]
